# Small Rab GTPases in Intracellular Vesicle Trafficking: The Case of Rab3A/Raphillin-3A Complex in the Kidney

**DOI:** 10.3390/ijms22147679

**Published:** 2021-07-18

**Authors:** Olga Martinez-Arroyo, Estela Selma-Soriano, Ana Ortega, Raquel Cortes, Josep Redon

**Affiliations:** 1 Cardiometabolic and Renal Risk Research Group, INCLIVA Biomedical Research Institute, 46010 Valencia, Spain; omartinez@incliva.es (O.M.-A.); raquel.cortes@uv.es (R.C.); 2 Physiopathology of Cellular and Organic Oxidative Stress Group, University of Valencia, 46100 Valencia, Spain; Estela.Selma@uv.es; 3 CIBERObn, Carlos III Institute, 28029 Madrid, Spain

**Keywords:** Rab proteins, Rab3A, Rabphilin-3A

## Abstract

Small Rab GTPases, the largest group of small monomeric GTPases, regulate vesicle trafficking in cells, which are integral to many cellular processes. Their role in neurological diseases, such as cancer and inflammation have been extensively studied, but their implication in kidney disease has not been researched in depth. Rab3a and its effector Rabphillin-3A (Rph3A) expression have been demonstrated to be present in the podocytes of normal kidneys of mice rats and humans, around vesicles contained in the foot processes, and they are overexpressed in diseases with proteinuria. In addition, the Rab3A knockout mice model induced profound cytoskeletal changes in podocytes of high glucose fed animals. Likewise, *RphA* interference in the *Drosophila* model produced structural and functional damage in nephrocytes with reduction in filtration capacities and nephrocyte number. Changes in the structure of cardiac fiber in the same *RphA*-interference model, open the question if *Rab3A* dysfunction would produce simultaneous damage in the heart and kidney cells, an attractive field that will require attention in the future.

## 1. Introduction

Small Rab GTPases, the largest group of small monomeric GTPases, and their Rab-interacting proteins, are involved in multiple cellular functions including the traffic of endosomal vesicles and plasma membrane combination to release cargoes to the extracellular space [1,2]. The role of Rab GTPases in diseases and specific syndromes has been broadly investigated [3]. Largely assessed and implicated in vesicle synapses and deposits in nervous system diseases, evidence of their association in other tissues and pathological processes such as cancer, inflammation, platelet, and endocrine secretions have been the motive of research in recent years.

Functionally, Rab GTPases coordinate membrane trafficking events on the basis of cyclic interconversion between active GTP-bound states and inactive GDP-bound states. The GTP-bound form can interact with effectors, promoting various steps and contributing to vectorial membrane traffic [2]. Each Rab protein has specific effector proteins that regulate distinct intracellular transport steps, which are the trans-Golgi network, endosomal pathway-lysosome for clearance/degradation, or fusion with the cellular membrance for vesicle release being the most studied [4,5,6].

Rabs are associated with inherited genetic and acquired diseases [7]. Their impact on cancer, neurologic disease, immunity, and infections have been recognized [8]. Despite the increase in knowledge, their contribution to kidney and heart dysfunction and damage is scarce. Therefore, this review provides an overview of the relevance of Rab GTPases in human diseases, with a special focus on the Rab3a-Rph3A complex and its critical role in kidney disease and a mention of future implications.

## 2. Rab GTPases and Vesicle Trafficking

Rab proteins [9] are master regulators of intracellular vesicle transport between different compartments through the recruitment of effectors and specific regulators [2], being involved in either vesicle budding, mobility through interaction with the cytoskeleton or tethering to the membrane. The Rab family is composed of more than 70 GTPases, each of which is preferentially associated with one intracellular compartment in order to control the specificity and directionality of membrane trafficking pathways, mostly related to vesicular transport. In doing so, they contribute to confer membrane identity [10] and ensure that membrane-bound cargoes are transported to their correct destinations within the cell.

**Figure 1:**
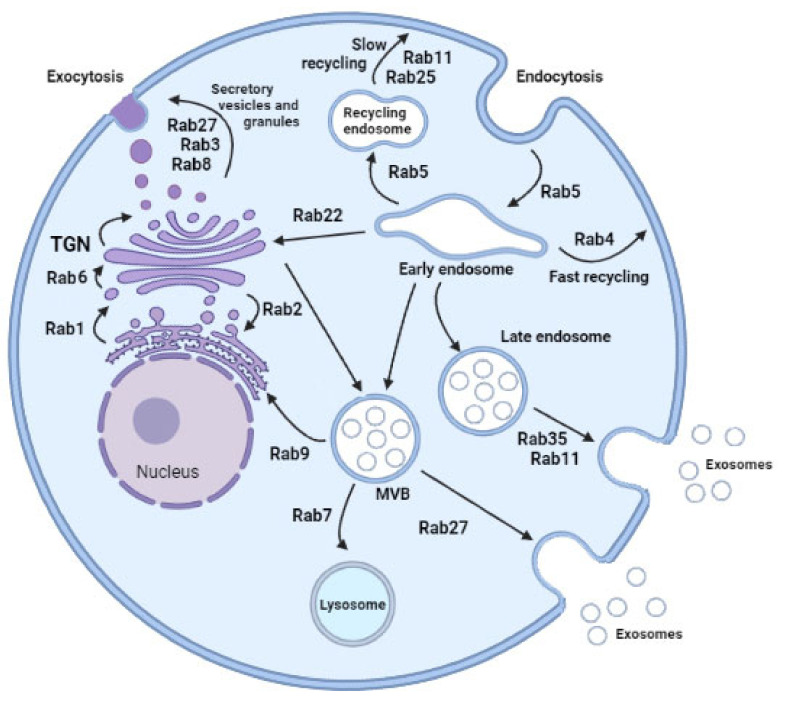
Rab proteins-mediated intracellular vesicular transport. Highlighted Rab family proteins that play key roles in regulating cellular membrane trafficking including endocytosis, exocytosis, exosome secretion, and vesicles delivery between organelles. Small Rab GTPases play key roles regulating cellular vesicular trafficking including endocytosis, exocytosis, exosome secretion, and intracellular vesicles delivery. Rab5, which is localized to early endosomes mediates endocytosis and endosome fusion of vesicles. Rab11 and Rab25 mediate slow endocytic recycling through recycling endosomes, whereas Rab4 mediates fast endocytic recycling directly from early endosomes. The late endosome-lysosome traffic associated Rab7 mediates maturation of late endosomes and their fusion with lysosomes. Another late endosomal GTPase, Rab9, mediates trafficking from late endosomes to the trans-Golgi network (TGN). Rab1, located at endoplasmic reticulum (ER) exit sites and the pre-Golgi intermediate compartment (IC), mediates ER–Golgi trafficking. Rab2, located at the IC, might also regulate Golgi–ER trafficking. Rab6 is mainly known for intra-Golgi trafficking of vesicles. Rab8 mediates constitutive trafficking from the TGN to the plasma membrane, while Rab3 and Rab27 mediate various types of regulated exocytic events. Rab22 mediates trafficking between the TGN and early endosomes and vice versa. Finally, for multivesicular body (MVB)-dependent secretion, Rab11, Rab35, and Rab27, have been shown to promote exosome secretion and may act on different MVBs along the endocytic pathway. MVB: multivesicular body; TGN: trans-Golgi network. Created in biorender.com.

The processes have well-defined trafficking routes, including exocytic and endocytic pathways, with the latter comprised of early, late, and recycling components [11]. Each of the pathways is usually embedded with non-overlapping Rabs and occur at different compartments (Figure 1).

Associated with the trans-Golgi network and secretory vesicles, several Rab proteins have been identified. Rab1 facilitates budding of vesicles at the endoplasmic reticulum exit site and the translocation of vesicles to the pre-Golgi intermediate compartment [12]. The transit of vesicles from the endoplasmic reticulum to pre-Golgi might also be regulated by Rab2-mediated reverse flow. Rab8 relays vesicle trafficking in the secretory route leading from the trans-Golgi network to the plasma membrane. Rab3 subfamily members are critical regulators of secretory vesicle exocytosis in eukaryotic cells, but recent studies indicate that additional Rab isoforms, Rab27 subfamily, are required for this process [13]. Recent literature reports that Rab3 and Rab27 cooperate to achieve vesicle exocytosis by recruiting specific effector proteins, such as Rabphilin3a (Rph3a) that bind Rab27a, recruiting an activating enzyme Rab3IL1, a Rab regulator, which catalyzes the exchange of GDP for GTP on Rab3a [14]. Rph3a drives vesicle docking at target membranes. However, some secretory vesicles. Rph3a drives vesicle docking at target membranes. However, some secretory vesicles probably also probably are formed through direct budding of the plasma membrane, and the requirement for Rab GTPase activity in such a secretion is not known.

In relation with endocytosis and recycling endosome pathways, Rab5 is a resident of early endosomes, phagosomes, plasma membranes, and mediates endocytosis involving clathrin-coated vesicles. Rab11 and Rab25 participate in the slow endosome recycling and Rab4 facilitates fast recycling of endosomes from early endosome populations. Then, Rab7 mediates maturation of late endosome (LE) and then ushers LE to the lysosome for degradation. Finally, Rab9 links LE to the trans-Golgi network. Focusing on exosome secretion, there are new molecular players in the pathogenesis of many diseases, Rab11 being the first reported [15]. Later, two new Rabs were emphasized in exosome secretion, Rab27 and Rab35, both allowing docking of multivesicular bodies (MVBs) to the plasma membrane. Silencing experiments of Rab2B, Rab5A, Rab9A, Rab11, and most efficiently Rab27A and Rab27B decreased secretion of exosomes [16,17,18]. However, these individual observations were different according to the cell types analyzed and influenced by exosome diversity enriched with different cargoes. Rab11 and Rab35 are associated mainly with recycling and early sorting endosomes, respectively, and Rab27A/B to late endosomal and secretory compartments, often called lysosome-related organelles [9]. Thus, different subtypes of late endosomes could generate different exosomes [19]. Perhaps exosome diversity in protein cargoes could be due to the different secretion traffic routes regulated by specific Rab proteins according to cell stimulus.

Moreover, Rab interactions with effectors regulate vesicle targeting and membrane fusion in three ways [20]. Firstly, Rabs help in the carriage of vesicles from their site of origin to the acceptor compartments through cytoskeleton elements and motor proteins. Secondly, Rab effectors regulate membrane trafficking at the vesicle docking step. Thirdly, in the membrane fusion of vesicles is SNAREs-driven docking on membrane [20]. In summary, the specific localization of different Rab GTPases to defined membrane compartments and their ability to regulate specific trafficking pathways, have made them attractive as novel molecular targets. However, there is partial knowledge about the precise function and impact of over- or under-expression of Rab GTPases on vesicle traffic derived of the heterogeneity of function in the different cell types with difficulties to identify their kinetics. Development of new technologies, such as high-resolution confocal microscopy [21] and CRISPR/Cas technology, contribute to obtaining solid information and a greater understanding of the role of Rab GTPases in vesicle biogenesis and secretion, revealing previously unknown physiological roles and therefore its impact on health and disease.

## 3. Rab-Mediated Vesicular Traffic and Diseases

The precise regulation of membrane trafficking processes by Rab GTPases is dependent on interactions with effectors, maintaining polarity of cells for achieving the maximal efficiency of function and structure and regulating cell signaling, division, survival, and migration [22,23]. Thus, their relevance in regulating the vesicle trafficking has led to investigating its relationship on the development of several diseases (Table 1).

How Rab proteins work and their role in disease has been extensively studied in cells of the nervous system, assessing the role on neuronal life cycle, activity, and intraneuronal deposits accumulation [24,25,26,27]. In different neurodegenerative disorders, changes and regulatory roles of Rab proteins in the pathological mechanisms in neurons, at the mitochondrial level, and glial cell dysfunctions have been investigated. In Alzheimer, Parkinson, Huntington Corea, Amyotrophic Lateral Sclerosis, and Charcot-Marie-Tooth diseases [25], alterations of Rab proteins have been identified. Although the Rab proteins alteration is not the primary cause of the disease in all of them, even being a consequence. Rab7, Rab28, and Rab11 mutations are the primary cause of Charcot-Marie-Tooth disease and Rab39B in Parkinson’s disease [28,29,30,31]. Secondary effects of RabGTPases on the membrane trafficking itself have been observed in many other diseases (See a wide review in 16). As an example on the complexity of mechanisms involved in the potential alterations of the Rab proteins are the studies performed on Parkinson’s disease [32]. Linked to Rab39b mutation, Rab29, Rab5a, and Rab7 have been identified in inherited early-onset Parkinson’s disease with Lewy’s body [31]. In contrast, Rab11 rescues several phenotypes, such as accumulation of Lewy protein [33]. Research in analyzing Rab and vesicular traffic mechanisms can help to understand what keeps neurons alive and the role in neurodegenerative disorders.

In cardiovascular diseases, relevant information has also been obtained. Increased expression of Rab1 in myocardium distorts subcellular localization of proteins and is sufficient to cause cardiac hypertrophy and failure [34]. In myocytes, Rab9-dependent autophagosomes recognize and engulf damaged mitochondria, resulting in ischemia-reperfusion (IR) changes in oxidative phosphorylation, eventually decreasing cardiomyocyte viability [35,36,37]. In diabetic patients with albuminuria, a genome-wide association study identified a genetic locus of Rab38 associated with albuminuria, highlighting novel pathways influencing albuminuria [38].

In platelets, their impact on haemostasis and thrombosis as well as on tissue regeneration, inflammation, and metastasis through uptake, packaging, and release of exosomes from storage vesicles are extensively studied due to their potential therapeutic implications [39]. The role in phagocytosis to engulf, kill, and process foreign bodies and apoptotic cells and presenting antigens to the immune cells have been identified as playing an important role in immune processes [40]. In IR injury, a phosphorylation-regulated polarization mechanism through Rab21 via Rph3A is important for neutrophil adhesion to endothelial cells during inflammatory responses [41]. In the liver, interferon regulatory factor 1 regulates Rab27a transcription and extracellular vesicle secretion, leading to oxidized phospholipid activation of neutrophils and subsequent hepatic IR injury [42].

**Table 1:** Primordial function and disease related to the most relevant Rab GTPases.

Rab Protein	Primordial Function	Diseases Related
	ENDOSOME RECYCLING	
Rab11	Slow transport endolysosomal vesicles from perinuclear recycling endosome compartment toward plasma membrane Three members: Rab11a, widely distributed; Rab11b, Rab 11 (Rab 25) restricted tissue expression pattern	Facilitate spread of colon cancer cells [33]
Rab35	Slow transport endolysosomal vesicles from perinuclear recycling endosome compartment toward plasma membrane Role not well established	
Rab4	Fast recycling after endocytosis	
Rab9	Transport to the Golgi network Autophagosome recognition and engulfing of damaged mitochondria.	Mitochondrial fission in cardiac myocytes. Eventually decreased cardiomyocyte viability [37]
Rab5A	Involved in exosome secretion regulatory pathways Fast delivery of cargo to the plasma membrane Overexpression inhibits progression of endocytosed material from early endosomes	
	ENDOSOME MATURATION	
Rab5ARab7	Interacts with 37 genes involved in exosome secretion regulatory pathways Maturation of late endosomes and their fusion with lysosomes Sorting and degradation Release Rab5	
	VESICLE SECRETION	
Rab27	Secretory protein Transport of late endosomal/lysosome-like compartments to the plasma membrane. Regulate exocytic events in a sequential manner together with Rab3 and Rab11	The first found to be involved in human disease. Important role in cancer progression and metastasis. High expression was associated with poor survival, lymph node, and distant metastasis [43] Increased in serum of diabetic patients [44]
Rab3	Secretory protein Regulate exocytic events	Renal dysfunction * Cardiac dysfunction *
Rab22A	Endosomal associated protein in different cell linesEctosome formation in other models	

* See Table 2.

Given the importance of Rab GTPases in membrane trafficking, and the relevance of this process for human health [22], it is perhaps surprising that only a limited number of genetic diseases are associated with Rab dysfunction. The existence of multiple Rab isoforms and trafficking pathways presumably makes humans less vulnerable to mutations in individual Rab-encoding genes.

**Table 2:** Primordial function and disease related of the most relevant Rab GTPase in the kidney.

Rab Protein	Primordial Function	Experimental Model	Diseases Related
Multiple	Transport endolysosomal vesicles	Multiple	
Rab5	Endocytosis Endocytic traffic of nephrin	Cultured podocyte *Drosophila* nephrocyte	SRNS with FGS by mutation in GAPVD1 and ANKFY1 genes [45]
Rab7	Protein degradation	*Drosophila* nephrocyte	
Rab11	Endocytic recycling	*Drosophila* nephrocyte	
Rab11b	Architectural structure Endocytic recycling Migration	Cultured podocyte Cultured fibroblast *Drosophila* nephrocyte	SRNS with FGS by mutation in TBC1D8B gene [46]
Rab3A	Architectural structure	Cultured podocyte *Drosophila* nephrocyte	Proteinuria [47,48]
Rab38	Endocytosis of albumin	Transgenic rats Cultured cells proximal tubule LLC-PK1)	Fawn-hooded hypertensive rat [49] Proteinuria [49]
Rab7	Maturation of late endosomes and their fusion with lysosomes Reduce activation of MMP-2 Aquaporine2 sorting in collecting duct cells	Cultured resident fibroblasts and tubular cells Collecting duct mpkCCD cells	Endothelial mesenchymal transition in diabetic nephropathy [50]
Rab27a	Cell polarization	Madin-Darby canine kidney II cells	Reduction of tight junction protein in tubular cells [51]
Rab27a	Reduce exosome release	Cultured podocyte Cultured renal tubular epithelial cells	Reduce inflammation of diabetic renal disease through the miR-26a-5p/CHAC1/NF-kB pathway [52]

SRNS with FGS steroid resistant nephrotic syndrome with focal glomeruloesclerosis. See text for further explanation.

### 3.1. Rab-Mediated Actions in Health and Disease in the Kidney

The kidneys are a crucial organ to maintain the homeostasis of volume fluids and electrolytes through the activity of specialized cells at different levels of the structure. Dysfunction of one of these produces a negative impact on the others, leading to a progressive reduction in renal function moving towards a Chronic Kidney Disease (CKD), which is highly prevalent in the general population, around 10%. Most cells in tissues and especially in the kidneys are polarized and usually have two distinct plasma membrane domains—an apical and a basolateral membrane—which are the result of polarized trafficking of proteins and lipids. In the renal glomerulus, podocytes are highly differentiated ramified cells that have a major role in the maintenance of the filtration barrier [53], in cooperation with the vascular endothelial and the mesangial cells. In the tubules, cells have multiple mechanisms specialized for different functions and those in the endothelial-mesenchymal translation play an important role in inflammatory mechanisms.

Knowledge about Rab GTPase in the different cellular components of the kidneys has been stablished, although the relevance in health and disease today is limited. Advances in understanding the role of Rab GTPase in the kidney has been obtained from assessing the expression and activity of the exocyst complex in the development and repairing of tubular damage, identified changes in Rab proteins induced by low-frequency genetic nephropathy or hypertension or assessing pathologic expression in models of proteinuria, both glomerular and tubular. Likewise, the role in exosome secretion was also examined. Culture renal cells and in vivo models have been used to search for the mechanisms in which the Rab proteins were involved. For example, nephrocytes of *Drosophila*, analog of podocytes and tubular cell at the same time, provide a tool to test Rab functions by using genetic manipulation. In Table 2, a summary of the knowledge about the Rab involvement in renal physiology and pathological states are presented.

The exocyst is a highly conserved eight-subunit protein complex involved in the targeting and docking of exocytic vesicles translocating from the trans-Golgi network to various sites in renal cells [54]. Many functions of exocyst in the kidney are the result of various small regulatory GTPases, being involved in primary ciliognesis, cystogenesis, and tubulogenesis. A role of repairing renal tubule epithelial cells after acute renal injury has also been recognized [55].

#### 3.1.1. Podocytes

Fu et al., identified 27 different Rabs in nephrocytes, eleven were functional and among them five were important to maintain functionality and three were essential. Silencing Rab5 identified their role in regulating endocytosis. While doing the same in Rab7 and Rab 11, changes in protein degradation and membrane recycling were observed. As a consequence of silencing these genes, disruption in the vesicles organization, integrity of filtration barrier, and subcellular structures in charge of protein reabsorption were observed [56].

Steroid-resistant nephrotic syndrome (SRNS) is a low frequency disease in which 30% of the cases in children are due to a gene mutation, with histological lesions of focal glomerulosclerosis (FSGS), a syndrome less frequent in adults. In missense mutations in the TBC1D8B gene (TBC1domain family), observed in two families with X-linked early-onset SRNS, decrease of Rab11b GTPase-activating protein, observing a podocyte with in the architectural changes and migration defects as result of lack of internalization and recycling processes [46]. Kamp et al., described five more families with this mutation and in the *Drosophila* model, confirmed the implication of Rab11 in the process [57]. Other mutations identified as causal of SRNS, were in the GAPVD1 gene, (endosomal regulator), and probably in the ANKFY1 gene (Ankyrin Repeat and FYVE Domain Containing 1). Both genes interacted with Rab5 and the mutations conversely increased binding to GTP-bound RAB5. The interaction of GAPVD1 and nephrin, a structural protein key in the slit diaphragm, suggests that endocytic trafficking of nephrin may play a role in an RAB5-mediated pathogenesis of the SRNS [45].

#### 3.1.2. Renal Tubules

Tubular functions, mainly those involved in protein reabsorption, are largely influenced by alteration in vesicular traffic. In the fawn-hooded hypertensive rat, a model of hypertension-associated renal disease, Rab38 affects urinary protein excretion in the proximal tubule. Using cultured proximal tubule LLC-PK1 cells, Rab38 mRNA knockdown reduced endocytosis of colloidal gold-coupled albumin [49]. Beside the role of Rab38 in the epithelial tubular cells, a relevant role of Rab7 has been also identified in protecting the development of endothelial mesenchymal transition (EMT) and apoptosis when albumin overload is induced. The overload of albumin in the tubular cell induced activation of MMP-2, matrix metalloproteinase-2, which degrades type IV collagen and reduces the thickening of the tubular basal membrane. Rab7 overexpression reduces the MMP-2 activity preserving the tubular basal membrane structure. The result of this study opens the possibility that activation of Rab7 will result in being useful to protect the impact of overload of proteins in the intratubular fluid reducing the EMT, such as occurs in diabetic nephropathy [50]. In addition, Slp2-a, an effector of Rab27A, regulates apical targeting of podocalyxin and its binding partner, ezrin, in concert with Rab27A. The authors established a model of claudin-2 expression through transport of podocalyxin to the apical surface by the function of the Slp2-a–Rab27A complex [51], which contributes to the amount of tight junction protein.

Lastly, the role of Rab proteins in the collecting tubule has been studied. Aquaporin is a vasopressin-regulated water channel protein responsible for osmotic water reabsorption by collecting ducts. In the presence of vasopressin, Rab7 and vacuolar protein sorting 35 (Vps35) participate in AQP2 sorting in early endosomes under vehicle conditions and apical membrane trafficking [58].

#### 3.1.3. Exosome Release

The impact of Rab proteins in exosome release has also been assessed with Rab27 playing a relevant role. Two recent studies identify that inhibiting or activating Rab27 expression results in a reduction or increment in exosome release from the podocytes. In the first of these studies, overexpression of KIBRA stabilized Rab27, avoiding its degradation via the ubiquitin-proteasome pathway [59]. In the second, in a model of inflammatory response to proximal tubular epithelial cells, knocking out Rab27a inhibited the excessive inflammatory response through the CHAC1/NF-κB pathway. This was due to the intracellular accumulation of the miR-26a, which thereby delayed the development of diabetic kidney disease [52].

### 3.2. Rab-3A/Rabphilin-3A Complex in Kidney Disease

In genetic association studies, our group identified SNPs related with the risk to develop microalbuminuria by using combined genetic and metabolomic data from a general population, finding an association between a polymorphism of the *RPH3A* gene and UAE [60]. Recently, in data from the Strong Heart Study a total of 277 variants annotated in genes *RPH3A, RPH3AL* (*Rabphilin 3A like*), *RAB3IL1* (RAB3A interacting protein like 1), and *RAB27A* were available for association analysis with UAE and GFR progression over time. Using mixed effects models to account for family relatedness and adjusting for potential confounders, polymorphisms at the *RAB3IL1* and *RPH3A* showed suggestive significant association with the progression of UAE and GFR levels (unpublished data). Furthermore, a previous study from Framingham cohort also showed an association between UAE and *RPH3A* [61].

In 2003, Rastaldi et al. investigated Rph3A and Rab3A as possible players in podocyte biology. Based on that Rph3A binds to Rab3A, it is a complex required for the docking of synaptic vesicles to their target membrane and that bind with cytoskeletal proteins in neurons, vesical and cytoskeletal are also important for podocyte homeostasis [47]. The complex Rab3A-Rph3A has been demonstrated in neurons and neuroendocrine structures as well as in normal kidneys from mice, rats, and humans in podocytes around vesicles contained in the foot processes [62,63]. In addition, in human biopsies of proteinuric patients with focal and segmental glomeruloesclerosis, an increment of Rab3A-Rph3A expression was also identified [47]. The potential role of these molecules in renal damage was further studied by the same group in a model of Rab3A knockout mice with high-glucose diet. The knockout mice developed altered podocyte actin plasticity with podocyte damage and proteinuria. Moreover, in the model with a high-glucose diet, renal lesions similar to those in human diabetic nephropathy were developed [64].

Our group in recent work has identified that Rph3A is expressed and colocalized with molecular markers of endocytic and exocytic pathways in pericardial nephrocytes of *Drosophila melanogaster*. Nephrocyte is a cell that combines podocyte and renal proximal tubule functions and is involved in the removal of waste products from the haemolymph and Malpighian tubules, a structure regarded as analogous to the renal tubular system [48]. There are two distinct nephrocyte populations: the garland cell nephrocytes around the esophagus (binucleate) and the pericardial nephrocytes (mononucleate) situated along the heart, both functionally, structurally, and molecularly similar to human podocytes [65].

**Figure 2:**
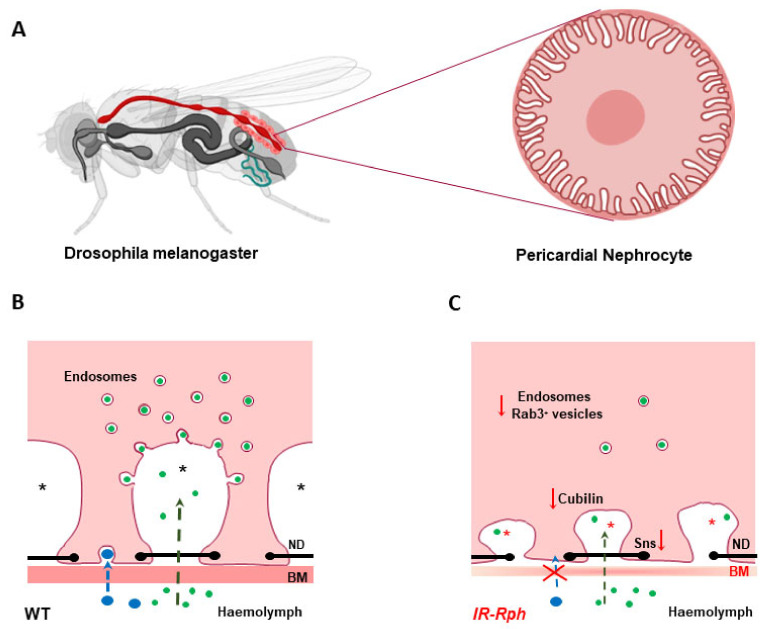
Schematic representation of nephrocyte alterations in Rph RNAi knockdown flies. (**A**) *Drosophila melanogaster* model and pericardial nephrocyte, respectively. (**B**,**C**), Schematic drawing of filtration and endocytosis in wild-type (**B**) and IR-Rph (**C**) nephrocytes. Knockdown of Rph seems to impair nephrocyte performance at two levels: filtration by basement membrane (BM) disruption (BM thinning) and subsequent cytoskeletal modifications leading to important structural alterations, such absence of labyrinthine channels, (asterisks in figure), and reduced expression of Stick and Stones (sns), ortholog to human nephrin protein, which mediates cell-cell recognition and adhesion in nephrocyte diaphragm (ND) (black line with dots)] and protein uptake by influencing Cubilin expression and vesicular trafficking (less endosomes number in IR-Rph). Small (green) and large (blue) dextran uptake in wild-type and Rph-RNAi knockdown pericardial cells. Created partly in biorender.com.

Several studies show that the endocytic pathway plays an important role in the development, maintenance, and damage of the podocyte and may lead to alterations in cell morphology [66,67,68]. Rph3A interference reduces the expression of Rab3a and the *Drosophila* ortholog of transcription factor Krüppel-like factor 15 (KLF15) is a zinc-finger transcription factor highly expressed in the glomeruli and interstitial cells. It has a protective role in proteinuric models, by reducing tubular and podocyte injury, renal fibrosis, and mesangial lesion. These reduced injury levels promote alteration in endocytic pathways that ultimately lose cell fate [69]. A morphological impact on nephrocytes was observed at several levels: (a) basement membrane disruption, and (b) cytoskeletal modifications leading to absence of labyrinthine channels and nephrocyte loss (Figure 2).

Rph3A interference also affects the expression of genes directly involved in the nephrocyte structure and function. Proteins Sns and Kirre, human nephrin, and neph1 orthologues, respectively, which interact through their extracellular domains to form the nephrocyte diaphragm [70], are reduced (silenced). The absence of Sns expression leads to a dramatic reduction of the nephrocyte number, concordant with recent studies which have documented the significance of cytoplasmic Sns in regulating intra-nephrocyte actin organization [71]. In addition, the Cubilin-Amnionless system, in charge of the endocytosis of small size proteins across the labyrinthine channels [72], is also affected by the Rph3A interference. Reduction in protein intake by the nephrocyte results from a decrease in Cubilin expression and vesicular trafficking affected by low levels of KLF15 [48] (Figure 2). Both systems, Sns proteins and Cubillin proteins, can be altered by a reduction in the gene expression or mis-localization of any of their components. Our study highlights the role of the reciprocal regulation between cytoskeletal components and nephrocyte diaphragm proteins as essential for size and charge-dependent filtration and provides data to support the previous results in the Rab3A knockout mice model, which induced proteinuria and renal insufficiency [64].

Simultaneously, our group has analyzed the impact of high glucose and angiotensin II on the Rab3A-Rph3A system in human immortalized podocytes and in urine cell pellets from patients with hypertension and diabetes [73]. Our results suggested that the Rab3A-Rph3A system could be involved in the alterations observed in injured podocytes and that a mechanism may be activated to reduce damage through the vesicular transport enhancement directed by this system. Finally, preliminary data from our group found that Rph3A is also expressed in the *Drosophila* myocardiocytes, and Rph3A silencing had a strong impact on the organization of fibers and functional cardiac parameters [74].

In summary, the Rab3A-Rph3A system plays a role in kidney damage mechanisms. Whether or not it could play a role in concomitant organ dysfunction, becomes an attractive field of research that requires attention in the future.

## 4. Conclusions

Rab-GTPases are proteins that mediate inter-compartmental transportation of vesicles carrying cargoes in and out of cells and organelles. Rabs have a predominant role in the regulation of vesicle trafficking in cells, which are integral to many cellular processes, such as cell proliferation, cell nutrition, innate immune response, mitosis, and apoptosis. Thus, Rabs are potential molecular targets in several diseases such as cancer, cardiovascular diseases, immunological disorders, and neurodegenerative diseases. However, the impact of altered Rabs expression is less understood in specific organs where vesicular trafficking has a high relevance on their structure and function, such as in the kidney. Recent studies have revealed the importance of the Rab3A-Rph3A complex in the morphology, cell loss, and filtration function in the kidney. Also, recently in the *Drosophila* heart, making it a new molecular player in the pathogenesis of renal and cardiac dysfunction and being a current field of interest with potential therapeutic applications. At this time, several questions about the exact mechanism of action remain unanswered and will require future research.
